# 1970. SARS-CoV-2 T-cell specific responses in allogeneic hematopoietic stem cell transplant recipients after second and third dose of BNT162b2 mRNA vaccine

**DOI:** 10.1093/ofid/ofac492.1595

**Published:** 2022-12-15

**Authors:** Iannetta Marco, Ilaria Spalliera, Flavia Mallegni, Lisa Mercante, Alessandra Imeneo, Angela Crea, Andrea Di Lorenzo, Laura Campogiani, Vincenzo Malagnino, Gottardo De Angelis, Raffaella Cerretti, Massimo Andreoni, Loredana Sarmati

**Affiliations:** University of Rome Tor Vergata, Rome, Lazio, Italy; Tor Vergata University of Rome, Rome, Lazio, Italy; Tor Vergata University, Rome, Lazio, Italy; Tor Vergata University, Rome, Lazio, Italy; Tor Vergata University of Rome, Rome, Lazio, Italy; Tor Vergata University of Rome, Rome, Lazio, Italy; Tor Vergata University of Rome, Rome, Lazio, Italy; Tor Vergata University of Rome, Rome, Lazio, Italy; Tor Vergata University of Rome, Rome, Lazio, Italy; Tor Vergata University of Rome, Rome, Lazio, Italy; Tor Vergata University of Rome, Rome, Lazio, Italy; Tor Vergata University of Rome, Rome, Lazio, Italy; Tor Vergata University of Rome, Rome, Lazio, Italy

## Abstract

**Background:**

Cell-mediated immunity (CMI) after anti-Severe Acute Respiratory Syndrome Coronavirus (SARS-CoV)-2 vaccines has been poorly explored in recipients of allogeneic hematopoietic stem-cell transplantation (HSCT), especially with regard to the 3^rd^ dose (booster).

Aim of the study was to assess the specific T-cell responses before and after the 3^rd^ dose of BNT162b2 mRNA vaccine in a cohort of allogeneic HSCT recipients, and compare it with healthy donors (HD).

**Methods:**

Allogenic HSCT recipients and HD were enrolled before receiving the 3^rd^ dose of BNT162b2 mRNA vaccine. Whole blood for T-cell specific responses was collected before (T1) and 8 weeks after (T2) the booster administration. T-cell responses were assessed with an Interferon (IFN)-γ release assay (IGRA), after overnight stimulation of heparin whole blood with pools of lyophilized peptides, covering the immunodominant sequence of the Spike (S) protein. IFN-γ production was assessed with an enzyme linked immunosorbent assay (ELISA). Statistical analysis was performed with GraphPadPrism.

**Results:**

14 HSCT recipients (8M, 6F) and 15 HD (7M, 8F) were enrolled (table 1). Median age was 47 [39-59] and 41 [31-48] years in the HSCT and HD groups, respectively.

Time between the vaccine 2^nd^ dose and T1 was significantly longer in HD than HSCT recipients (p< .001), while the time between T1 and T2 did not differ between the two groups.

SARS-CoV-2 S specific T-cell responses at T1 were inferior in HSCT recipients compared to HD (median IFN-γ production: 463 vs 231 ng/ml, respectively), although the difference did not reach the statistical significance. No differences were observed at T2. In a before-after analysis, SARS-CoV-2 S specific T-cell responses were significantly increased in HSCT recipients at T2 compared to T1 (median IFN-γ production: 267 vs 881 ng/ml, p=0.02) (Figure 1). At T1, 3 HSCT recipients showed very low or no IFN-γ production, while at T2 only 1 patient still had undetectable IFN-γ production after S peptide stimulation.

**Table 1:**
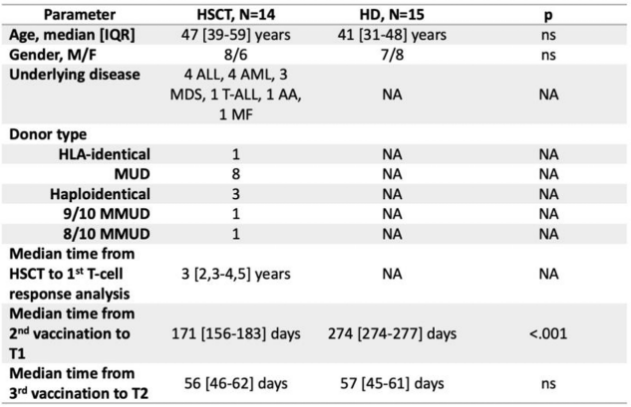
Clinical characteristics of allo-HSCT recipients and HD

**Figure 1:**
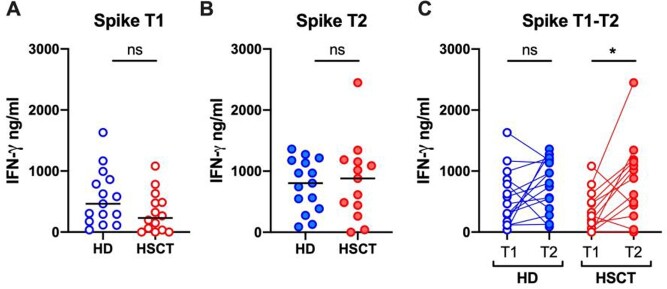
IFN-γ Production after Spike peptide stimulation of whole blood from allogenic HSCT recipients and HD.

**Conclusion:**

SARS-CoV-2 IGRA represents a useful tool to assess CMI, also in immunocompromised hosts. An additional 3^rd^ BNT162b2 mRNA vaccine booster dose seems to enhance CMI in allogenic HSCT recipients. Further studies are needed to evaluate the duration of SARS-CoV-2 CMI in HSCT recipients compared to HD.

**Disclosures:**

**All Authors**: No reported disclosures.

**Table 1:** Clinical characteristics of allo-HSCT recipients and HD

HSCT: hematopoietic stem-cell transplantation; IQR: interquartile range; ALL: acute lymphoblastic leukemia; AML: acute myeloid leukemia; MDS: myelodysplastic syndrome; T-ALL: T-cell acute lymphoblastic leukemia; AA: aplastic anemia; PMF: primary myelofibrosis; MUD: matched unrelated donor; MMUD: mismatched unrelated donors; NA: not applicable; ns: not significant.

**Figure 1:** IFN-γ Production after Spike peptide stimulation of whole blood from allogenic HSCT recipients and HD.

Comparison of IFN-γ production after Spike peptide stimulation of whole blood in allogenic HSCT recipients and HD at T1 (1.A) and T2 (1.B) (before and after BNT162b2 mRNA vaccine 3rd dose, respectively). Before-after representation of IFN-γ production after Spike peptide stimulation in HD and allogenic HSCT recipients (1.C).

